# Defining the reference range for right ventricular systolic strain by echocardiography in healthy subjects: A meta-analysis

**DOI:** 10.1371/journal.pone.0256547

**Published:** 2021-08-20

**Authors:** Tom Kai Ming Wang, Richard A. Grimm, L. Leonardo Rodriguez, Patrick Collier, Brian P. Griffin, Zoran B. Popović

**Affiliations:** Section of Cardiovascular Imaging, Heart, Vascular and Thoracic Institute, Cleveland Clinic, Cleveland, Ohio, United States of America; Wayne State University, UNITED STATES

## Abstract

**Background:**

Right ventricular (RV) systolic strain has recently demonstrated prognostic value in various cardiovascular diseases. Despite this, the reference range including the lower limit of normal (LLN) and factors associated with RV strain measurements are not well-established. This meta-analysis aimed to determine the mean and LLN of two- (2D) and three-dimensional (3D) right ventricular global (RVGLS), free wall (RVFWLS) and interventricular septal wall (IVSLS) longitudinal strains in healthy individuals and factors that affect strain measurements.

**Methods:**

In this meta-analysis, Pubmed, Embase and Cochrane databases were searched until 31 July 2020 for eligible studies reporting RVGLS, RVFWLS and/or IVSLS in at least 30 healthy subjects. We pooled the means and LLNs of RV strains by two- (2D) and three- (3D) dimensional echocardiography, and performed meta-regression analyses.

**Results:**

From 788 articles screened, 45 eligible studies totaling 4439 healthy subjects were eligible for analysis. Pooled means and LLNs with 95% confidence intervals for 2D- RV strains were RVGLS -23.4% (-24.2%, -22.6%) and -16.4% (-17.3%, -15.5%) in 27 studies; RVFWLS -26.9% (-28.0%, -25.9%) and -18.0% (-19.2%, -16.9%) in 32 studies; and IVSLS –20.4% (-22.0%, -18.9%) and -11.5% (-13.6%, -9.6%) in 10 studies, and similar results for 3D- RV strains. Right ventricular fractional area change and vendor software were associated with 2D-RVGLS and RVFWLS means and LLNs.

**Conclusion:**

We reported the pooled means and LLNs of RV systolic strains in healthy subjects, to define thresholds for abnormal, borderline and normal strains. Important factors associated with RV systolic strains include right ventricular fractional area change and vendor software.

## Introduction

Strain measured by speckle-tracking echocardiography has become an indispensable method for chamber quantification. While most research has been dedicated to the utility of left ventricular global systolic strain [1–3], several studies demonstrated the usefulness of right ventricular (RV) systolic strain in a variety of clinical scenarios [4–6]. The three main parameters of RV strain are right ventricular global longitudinal strain (RVGLS), right ventricular free wall longitudinal strain (RVFWLS) and interventricular septal wall longitudinal strain (IVSLS). However, the reference range for these parameters are still not established [1, 7]. The single previous meta-analysis for RV strains in healthy individuals did not report the pooled estimates of the lower limit of normal (LLN) of RV strain to define the reference range, did not separate RV strain obtained by two-dimensional (2D-) and three-dimensional (3D-) echocardiography techniques, nor adjust for potential confounders by meta-regression, and was limited by relatively modest number of subjects (n = 486) [8]. In other words, its results cannot be used to separate normal from abnormal RV strain values. While current echocardiography guidelines try to overcome this by recommending -20% as the abnormality threshold for RVFWLS, they acknowledge that this threshold was consensus-based on limited data and may vary based on factors such as vendor software [1].

Recently, we performed meta-analysis to not only provide the point estimate of average value, but also the reference ranges for normal values of left ventricle strain parameters obtained by 2- and 3-dimensional echocardiography along with magnetic resonance imaging for the first time [9, 10]. This meta-analysis aims to pool the means and LLN of two-dimensional RVGLS and RVFWLS parameters measured by echocardiography in healthy subjects to determine reference ranges. In addition, we assessed clinical and echocardiographic characteristics that influence RV strain parameters using meta-regression.

## Materials and methods

We followed the PRISMA guidelines for this meta-analysis, with a supplementary information file checklist provided and no separate protocol. The inclusion criteria were that studies must report original data for either RVGLS and/or RVFWLS measured by speckle-tracking echocardiography as the mean ± (standard deviation (SD), standard error (SE) or 95% confidence interval (95%CI)), or median (lower quartile, upper quartile) for at least 30 healthy adult subjects, with males and females both constituting at least one third of the individual study cohorts, especially for case-control studies where certain cardiovascular disease may favor one gender over another, to maximize applicability to the general population with equal split [11]. Healthy subjects from both cohort studies and control groups of case-control studies can be included. Studies including those with known cardiovascular disease, risk factors such as diabetes and hypertension, chronic single or multi-organ disease or malignancy, on cardiac medications or with abnormal cardiac examination or investigations were excluded. These should be stated in the methods section of individual studies and/or their absence demonstrated in baseline characteristics descriptions and tables to be eligible as healthy patients and studies. Studies measuring strain other than by speckle-tracking were excluded.

We searched the Pubmed, Cochrane and Embase databases through to 30 September 2020 using the search terms “(right) AND (ventricle) AND (strain) AND (echocardiography)” and with human subjects and adults (19+ years) as filter, and no language filters. Reference lists of related articles were assessed. After screening all abstracts from the search results, potentially suitable studies had their full-text articles carefully reviewed for final eligibility. Reviews, case reports, editorials, guidelines, conference abstracts and unpublished data were excluded. Amongst studies published by the same author group, we only chose the largest study and assumed the others contained overlapping healthy subjects as duplicates unless specifically stated in their methods that there were mutually exclusive cohorts.

From eligible studies, data extracted into include author surname, year, number of healthy subjects, country, disease studied, age, sex, body mass index, body surface area, heart rate, systolic blood pressure, echocardiography machine, vendor software, frame rate, 2D- or 3D- echocardiography, left ventricular ejection fraction, left ventricular global longitudinal strain, right ventricular basal diameter, right ventricular fractional area change (RVFAC), right ventricular lateral annular tissue Doppler S’ velocity, tricuspid annular plane systolic excursion and right ventricular systolic pressure. The strain outcomes of interest collected were RVGLS, RVFWLS, IVSLS, and the strain rate for all three right ventricular longitudinal strains. Authors TKMW and ZP screened studies and reached consensus for inclusion, and TKMW extracted study data for analyses.

Pooled analyses for healthy subjects from eligible studies to calculate the mean and lower limit of normal were separately performed for each of the three types of RV strains, and RV strain rates, and by two or three-dimensional echocardiography, if reported in healthy subjects by three or more studies. Using the mean and standard deviation of RV parameter in each study, the LLN is defined as the lower magnitude boundary of the 95%CI for the RV parameter (ie 1.96 times standard deviation from the mean). The standard error of the LLN (ie SE_LLN_) can then be derived from the standard deviation of the sample mean (ie SD_mean_) and the number of patients in the sample (n) using Bland’s proposed formula [12]: SE_LLN_ = √(SD_mean_^2^×(1/n+2/(n—1)).

Based on the mean and LLN with their SD or SE, meta-analysis was performed using the DerSimonian-Laird method random effects models to estimate pooled RV strain parameter means and LLNs with 95%CIs, and Forest Plots presented in all eligible studies reporting the RV strain parameters analyzed [9, 10]. These analyses were also performed for purpose of sensitivity analysis performed for cohort studies of healthy subjects only and excluding case-controlled studies, and performed separately by strain vendor software if reported in at least 2 studies due to presumptive differences. The associations between clinical and echocardiographic characteristics with RVGLS and RVFWLS means and LLNs were assessed using univariable meta-regression to report beta coefficients with 95%CI and P-value first in studies, and then for sensitivity analysis purpose in studies using the GE EchoPAC strain vendor software only. Pre-specified reference groups for some categorical variables include other Asian versus non-Asian countries for country, GE for echocardiography machine and GE EchoPAC versus non GE Echo PAC for vendor software. Vendor software was pre-specified for subgroup analyses of pooled RVGLS and RVFWLS. Quality of studies and risk of bias was evaluated using the Newcastle-Ottawa quality assessment scale for case control studies. In studies that compared RV strain data between subjects with disease and control (i.e. reference) sample, this quality assessment scale has a range of 0 to 8, with 8 being the optimal quality. In studies of healthy subjects only of the quality assessment scale had a range of 0 to 2 as the criteria referring to “cases” and “exposure” are not applicable. Study heterogeneity and publication bias were also assessed. The Cochrane Q test (P-value) and I^2 (inconsistency) statistic were used to evaluate the heterogeneity of studies. Funnel plots were used to assess for publication bias. All meta-analyses were performed with OpenMeta-Analyst software [13], and P<0.05 was deemed to be statistically significant.

## Results

**Fig 1:**
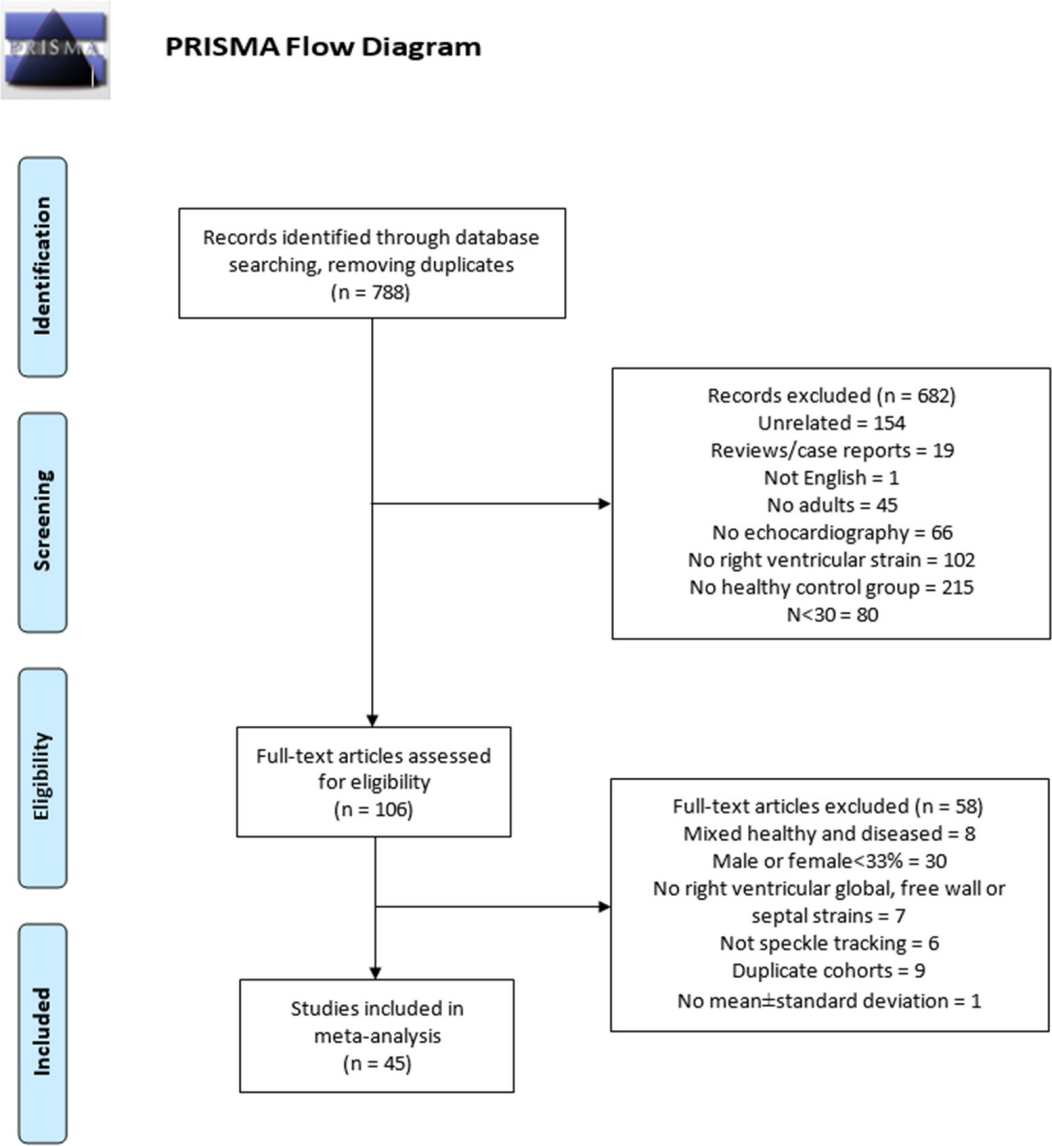
PRISMA diagram from literature search to eligible studies.

Disposition of studies from the literature search are shown in Fig 1. There were 788 articles obtained from the literature search, and after screening all abstracts 106 full-text articles were reviewed, identifying 45 eligible studies and 4439 healthy subjects for analyses [8, 14–54]. Clinical and study characteristics of eligible studies are listed in Table 1. Studies were published between 2009–2020, with 30–493 subjects, with 30 case-controlled studies and 15 cohort studies of only healthy subjects. The range for mean age was 23–67 years old and the range for male gender was 33–66%. Echocardiographic characteristics of eligible studies are listed in Table 2. There were 43 studies reporting RV strain by 2D- and 3 studies by 3D- echocardiography (1 reported both). Most studies had high study quality as assessed by the Newcastle-Ottawa quality assessment scale, with the most common unmet criteria being control selection, where there were no clear indication if subjects were selected as volunteers from the community (20/45 studies), and comparability, where the controls were not matched in some way to the cases (16 of 30 case-control studies).

**Table 1:** Characteristics of eligible studies–study and clinical factors.

Author	Year	N	Country	Disease studied	Study quality	Age (years)	Male (%)	BMI (kg/m^2^)	BSA (m^2^)	HR (/min)	SBP (mmHg)
Addetia	2016	259	US	None	2/8	44±15	54%		1.7±0.3	63	
Barbosa	2014	38	Brazil	Chagas disease	7/8	44±9	58%		1.8±0.2	66±9	126±15
Becker	2010	31	Germany	Transposition of great arteries repaired	7/8	23±3	45%			69±6	128±5
Berceanu	2019	90	Romania	Type 1 diabetes	6/8	30±8	66%	23±4	1.8±0.2	81±14	117±8
Bostan	2020	70	Turkey	Smokers	7/8	34±10	63%		1.6±0.3	76±11	123±7
Cai	2017	37	US	Stress /ischemic cardiomyopathy	7/8	62±13	41%				
Cappelli	2012	31	Italy	AL amyloidosis	8/8	67±5					
Chia	2014	142	Australia	None	2/2	45±15	53%		1.8±0.2	77±11	118±11
Clemmensen	2016	41	Denmark	Heart transplant	6/8	51±12	59%	24±2		62±7	
D’Andrea	2016	45	Italy	Pulmonary fibrosis	7/8	65±8	51%	30±4		78±7	135±8
Di Stefano	2020	97	US	Cardiac sarcoidosis	6/8	40±14	63%	26±5		73±13	116±16
Durmus	2015	40	Turkey	Systemic sclerosis	8/8	46±8	48%				
Fine	2013	186	US	None	1/2	44±16	39%	25±5	1.9±0.2	72±12	112±16
Fine	2015	116	US	None	1/2	48±16	42%	26±4		70±12	117±13
Forsha	2014	40	Denmark	None	2/2	29 (18–52 range)	40%				
Gudendouz	2012	39	France	Heart failure	8/8	50±15	54%	25±5		68±11	137±6
Haeck	2012	30	Netherlands	Pulmonary hypertension	6/8	53±12	33%		1.9±0.2		
Ichkawa	2013	33	Japan	Pulmonary hypertension	7/8	57±14	45%	22±3		63±10	117±12
Jategaonkar	2009	34	Germany	Atrial septal defect	7/8	47±9	62%				
Kanar	2018	41	Turkey	Chronic obstructive lung disease	7/8	58±10	53%	31±8		85±18	
Khan	2018	50	US	Pulmonary emboli	7/8	57±17	34%		1.9±0.2		126±17
Kurt	2012	34	Turkey	None	1/2	36±9.2	53%	25±4	1.8±0.1	80±12	117±23
Lai	2017	70	China	Tetralogy of Fallot repaired	6/8	23±6	57%	21			
Lakatos	2018	40	Hungary	Athlete’s heart	7/8	20±3	50%	22±3	1.8±0.2	76±17	116±16
Li	2017	30	China	Left bundle branch block	7/8	58±8	47%		1.7±0.2	74±10	124±13
Lindqvist	2006	30	Sweden	None	1/2	60±11	40%	25		62±9	135±16
McGhie	2017	155	Netherlands	None	1/2	45±14	50%	24±3	1.9±0.2	62±10	127±15
Menting	2015	85	Netherlands	Tetralogy of Fallot repaired	7/8	34±12	56%	24±3			125±13
Meris	2010	100	Switzerland	Right ventricle dysfunction	7/8	43±3	54%				
Morris	2016	238	Germany	Heart failure	6/8	37±13	50%	23±2			119±10
Muraru	2016	276	Italy	None	2/2	44 (32–56 IQR)	45%	23 (21–25 IQR)	1.8±0.2	65 (60–73 IQR)	120 (110–130 IQR)
Nel	2020	253	United Kingdom	None	2/2	36±12	41%	28±6	1.8±0.2	76±12	121±12
Park	2017	493	Korea	None	2/2	47±15	47%	23±3	1.7±0.2	68±10	120±12
Platz	2012	30	US	Pulmonary emboli	6/8	50±15	43%			71±15	
Rimbas	2020	151	Romania and Italy	None	2/2	51±14	59%	25±3			121±14
Rosca	2018	30	Romania	Hypertrophic cardiomyopathy	8/8	48±9	40%	25±3			
Roushdy	2016	30	Egypt	Ballooned mitral stenosis	7/8						
Sanz-de La Garza	2019	80	Spain	None	2/2	37±5	50%		1.8±0.2	65±10	117±12
Sarariva	2019	77	Brazil	None	1/2	40±10	47%	25±3			122±12
Schieirlynck	2020	82	Brussels	Brugada syndrome	8/8	48 (33–55)	51%				
Serrano-Ferrer	2014	40	Australia	Metabolic syndrome	7/8	58±4	50%	24±3		61±4	116±11
Smith	2014	60	UK	Pulmonary hypertension	7/8	41±12	40%				
Tadic	2015.1	54	Italy	Diabetes mellitus	7/8	51±8	54%	24±3	1.9±0.1	74±7	122±11
Vitarelli	2015	30	Italy	Obstructive sleep apnea	6/8	46±13	37%	26±4	1.8±0.2	69±9	120±8
Yoshida	2019	481	Japan	None	1/2	60±12	46%	22±2			113±12

N = number of patients, BMI = body mass index, BSA = body surface area, HR = heart rate, SBP = systolic blood pressure, DBP = diastolic blood pressure, US = United States, ± = mean±standard deviation, IQR = interquartile range. Study quality and risk of bias was assessed using the Newcastle-Ottawa quality assessment scale for case-controlled studies, out of 8 for those which studied diseases and controls, and out of 2 for those who only studied healthy subject controls, as explained in the methods section.

**Table 2:** Characteristics of eligible studies–echocardiographic factors.

Author	Year	N	Machine	Vendor	Frame rate (Hz)	2D/3D	LVEF (%)	LVGLS (%)	RVD (mm)	RVFAC (%)	RVS’ (cm/s)	TAPSE (cm)	RVSP (mmHg)
Addetia	2016	259	Phillips	Epsilon		2D	62±6	-18±2		37±4			
Barbosa	2014	38	GE	EchoPAC		2D	70±6	-21±2			11±2		24±3
Becker	2010	31	GE	EchoPAC		2D		-17±4					
Berceanu	2019	90	GE	EchoPAC		2D	58±4			46±6		22±4	
Bostan	2020	70	Phillips	Qlab	>50	2D	55±4	-21±2	31±5	41±8	15±2	24±3	
Cai	2017	37	Phillips	Epsilon		2D	61±5		37±6		14±3	22±4	
Cappelli	2012	31	GE	EchoPAC	80–100	2D	62±5	-19±7	26±4		13±2	24±4	25±5
Chia	2014	142	GE	EchoPAC	>60	2D	59±8		32±4	42±6	12±2	22±2	21±7
Clemmensen	2016	41	GE	EchoPAC	>55	2D					12±2	27±4	21±5
D’Andrea	2016	45	Esoate	EchoPAC	60–90	2D	57±4	-19±3	31±4		12±6	23±3	25±4
Di Stefano	2020	97	GE, Philips	TomTec	40–90	2D	64±4	-18±3					
Durmus	2015	40	GE	EchoPAC		2D	65±5	-21±3	27±3	34±4	13±2	24±3	20±6
Fine	2013	186	GE, Phillips, Siemens	VVI	40–90	2D	63±4	-17±3					24±7
Fine	2015	116	GE	EchoPAC	77±8	2D	63±5			46±1	14±2	24±4	28±5
Forsha	2014	40	GE	EchoPAC	50–90	3D	59±4			43±5		22±3	
Gudendouz	2012	39	GE	EchoPAC	>50	2D	59±9	-20±3		49±5	14±2	25±4	
Haeck	2012	30	GE	EchoPAC	>40	2D	62±7		30±3	37±9		23±3	21±3
Ichkawa	2013	33	GE	EchoPAC	76±15	2D	66±5	-20±2		52±5			
Jategaonkar	2009	34	GE	EchoPAC	40–80	2D			26±4			19±6	
Kanar	2018	41	Phillips		70–80	2D	61±5		28±2	54±4		20±3	21±8
Khan	2018	50	GE, Phillips, Toshiba	VVI		2D				20±9			26±6
Kurt	2012	34	GE	EchoPAC	50–90	2D	63±3						
Lai	2017	70	GE	TomTec		2D	57±4	-18±3					
Lakatos	2018	40	Phillips	TomTec		2D/3D	63±3		30±4	53±6	13±2	24±4	
Li	2017	30	GE	EchoPAC	57–72	2D	64±5		32±3	56±5	12±2	23±2	
Lindqvist	2006	30	GE	EchoPAC	86±11	2D	59±8						
McGhie	2017	155	Phillips	TomTec		2D			38±5	43±8	12±2	26±4	
Menting	2015	85	Phillips	Qlab		2D	58±5		37±6	45±8		28±4	
Meris	2010	100	GE	EchoPAC	70–80	2D	66±5		30±3	45±6		25±4	
Morris	2016	238	GE	EchoPAC		2D	63±6	-21±2		49±8	13±2	20±3	
Muraru	2016	276	GE	EchoPAC		2D				49±6		25 (23, 27 IQR)	21 (18, 26 IQR)
Nel	2020	253	Philips	Qlab	>50	2D	62±6		31±5	42±6		22±3	
Park	2017	493	GE	EchoPAC	60	2D	62±4		34±4	48±6		23±3	
Platz	2012	30	GE, Philips, Siemens	VVI	40±11	2D	>55						
Rimbas	2020	151	GE	EchoPAC		2D	60±7	-21±3		47±9		25±4	22±8
Rosca	2018	30	GE	EchoPAC	60–80	2D	61±4	-21±2	30±3	48±7	14±2	24±3	23±3
Roushdy	2016	30	GE		50–90	2D	57±7	-21±2		45±8		24±3	
Sanz-de La Garza	2019	80	GE	EchoPAC	60–80	2D	57±5			48±6	10±1	23±3	
Sarariva	2019	77	GE	EchoPAC		2D	68±6				15±2	24±4	26±5
Schieirlynck	2020	82	GE	EchoPAC		2D	63±6	-19±2		44 (36–50)		24±4	
Serrano-Ferrer	2014	40	Esaote	VVI	>60	2D	64±5		39±5		14±2	25±2	
Smith	2014	60	Toshiba	Aplio Artida		3D						24±4	26±4
Tadic	2015	54	GE	EchoPAC		2D	65±4	-21±2	20±4		13±2	22±3	20±4
Vitarelli	2015	30	GE	EchoPAC		2D	61±6			49±12	13±3	23±6	22±3
Yoshida	2019	481	Toshiba	TomTec		2D	64±5	-22±3		45±8			

N = number of patients, 2D = two-dimensional, 3D = three-dimensional, VVI = velocity vector imaging, LVEF = left ventricular ejection fraction, LVGLS = left ventricular global longitudinal strain, RVD+right ventricular basal diameter, RVFAC = right ventricular fractional area change, RVS’ = right ventricular S’ by tissue Doppler, TAPSE = tricuspid annular plane systolic excursion, RVSP = right ventricular systolic pressure, ± = mean±standard deviation, IQR = interquartile range.

**Fig 2:**
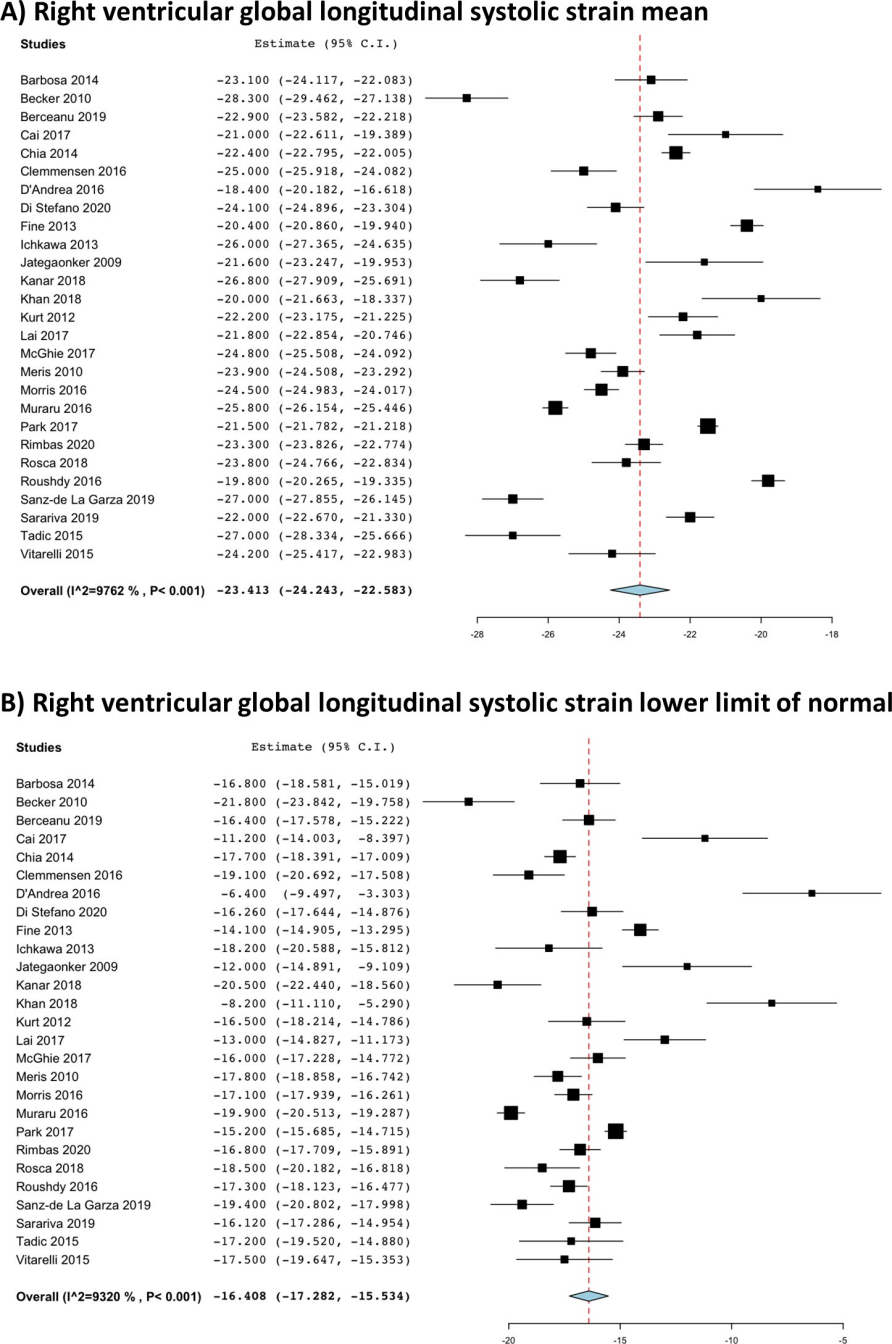
Right ventricular two-dimensional global longitudinal strain pooled (a) mean and (b) lower limit of normal.

**Fig 3:**
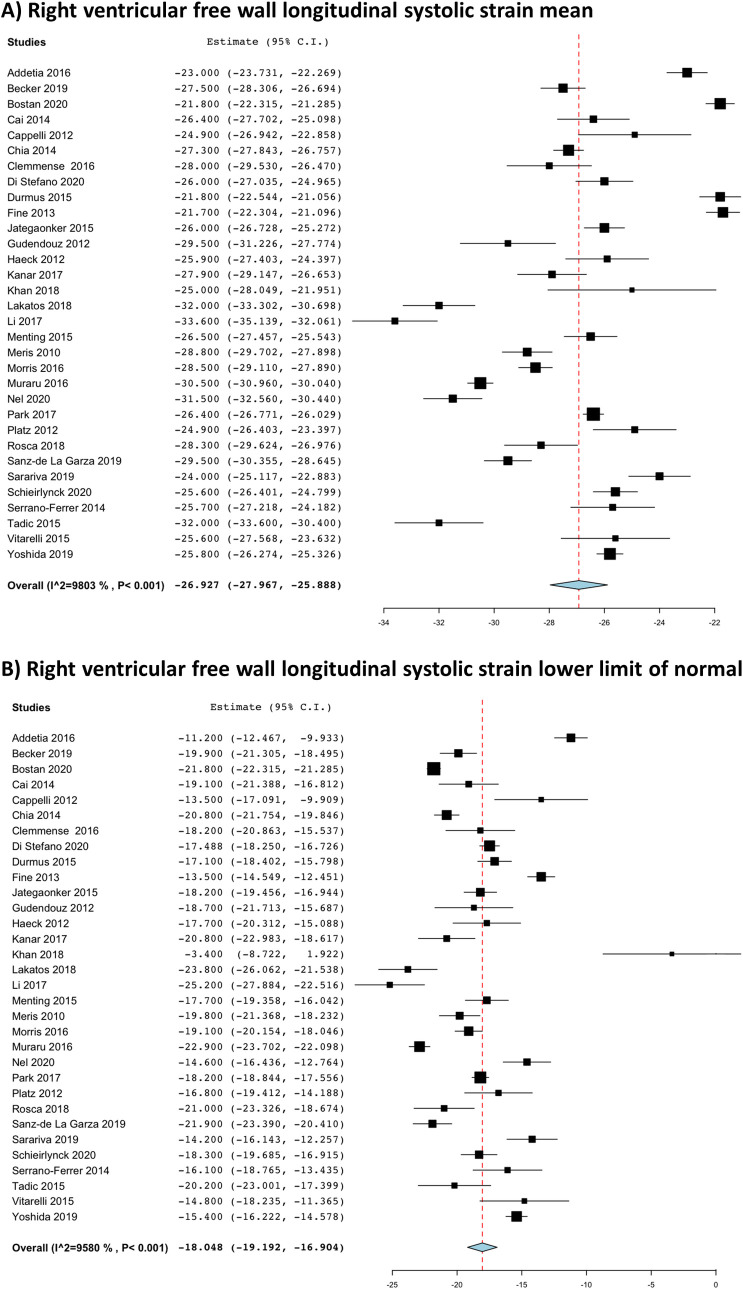
Right ventricular two-dimensional free wall longitudinal strain pooled (a) mean and (b) lower limit of normal.

**Fig 4:**
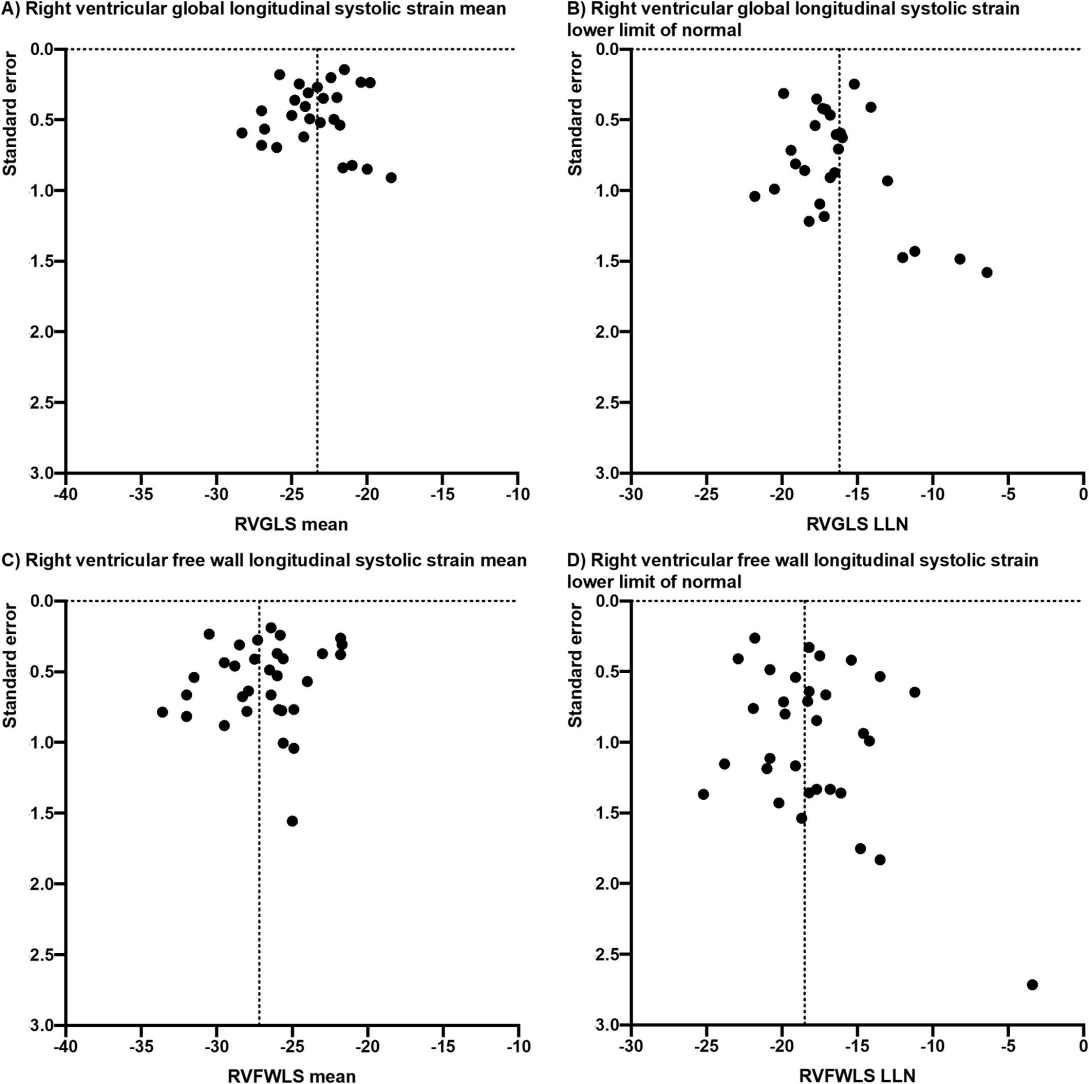
Funnel plots for pooled analysis (a) right ventricular global longitudinal strain (RVGLS) mean, (b) RVGLS lower limit of normal (LLN), (c) right ventricular free wall longitudinal strain (RVFWLS) mean, and (d) RVFWLS LLN.

Pooled means and LLNs for 2D- and 3D- RV strains are presented in Table 3. The pooled means and LLNs (95%Cis) for 2D- RV strains were RVGLS -23.4% (-24.2%, -22.6%) and -16.4% (-17.3%, -15.5%) in 27 studies (Fig 2); RVFWLS -26.9% (-28.0%, -25.9%) and -18.0% (-19.2%, -16.9%) in 32 studies (Fig 3); and IVSLS -20.4% (-22.0%, -189%) and -11.5% (-13.6%, -9.6%) in 10 studies. Significant heterogeneity was observed in all of these pooled analyses, but no significant publication bias was seen with symmetrical Funnel plots demonstrated in Fig 4. Separate pooled analyses of 2D-RVGLS and RVFWLS means and LLNs by various strain vendor software is shown in S1 Table in S1 Appendix, where velocity vector imaging technique appeared to have lower mean and LLN strain magnitude than other vendors. Sensitivity analyses of pooling only cohort studies with healthy subject and excluding case-controlled studies are displayed in S2 Table in S1 Appendix, which showed similar findings to the pooling all studies in Table 3.

**Table 3:** Pooled means and lower limits of normal for right ventricular strain.

Strain	Studies	N	Mean	95%CI (mean)	Heterogeneity testing	LLN	95%CI (LLN)	Heterogeneity testing
**2D**								
RVGLS (%)	27	2674	-23.4%	-24.2%, -22.6%	1091 (<0.001), 97.6%	-16.4%	-17.3%, -15.5%	382 (<0.001), 93.2%
RVFWLS (%)	32	3673	-26.9%	-28.0%, -25.9%	1575 (<0.001), 98.0%	-18.0%	-19.2%, -16.9%	737 (<0.001), 95.8%
IVSLS (%)	10	1230	-20.4%	-22.0%, -18.9%	425 (<0.001), 97.8%	-11.5%	-13.6%, -9.6%	223 (<0.001), 96.0%
RVGLSR (/s)	10	1134	-1.45	-1.59, -1.31	593 (<0.001), 98.5%	-0.80	-0.92, -0.67	143 (<0.001), 93.7%
RVFWLSR (/s)	6	954	-1.58	-1.88, -1.29	684 (<0.001), 99.3%	-0.84	-1.09, -0.59	168 (<0.001), 97.0%
IVSLSR (/s)	4	763	-1.27	-1.48, -1.07	224 (<0.001), 98.7%	-0.63	-0.88, -0.38	116 (<0.001), 97.4%
**3D**								
RVGLS (%)	3	140	-21.3%	-24.6%, -17.9%	71 (<0.001), 95.8%	-13.9%	-20.6%, -7.3%	80 (<0.001), 97.5%
RVFWLS (%)	2	80	-25.5%	-28.6%, -22.3%	9 (0.003), 88.7%	-16.2%	-22.3%, -10.1%	11 (<0.001), 90.8%
IVSLS (%)	2	80	-20.8%	-22.6%, -18.9%	4 (0.050), 73.9%	-13.5%	-19.0%, -8.0%	11 (<0.001), 90.9%

95%CI = 95% confidence interval, LLN = lower limit of normal, 2D = two-dimensional, 3D = three-dimensional, RV = right ventricle, GLS = global longitudinal strain, FWLS = free wall longitudinal strain, IVSLS = interventricular septal longitudinal strain, LSR = longitudinal strain rate, Heterogeneity testing include the Cochrane Q (P-value) and I^2.

Corresponding results for 3D-RV strains as shown in Table 3 were RVGLS -21.8% (-24.1%, -19.5%) and -15.2% (-19.6%, -10.8%) in 4 studies; RVFWLS -26.2% (-28.3%, -24.1%) and -17.6% (-21.6%, -13.6%) in 3 studies; and IVSLS -20.1% (-21.0%, -19.2%), and -14.6% (-16.8%, -12.5%) in 3 studies. Significant heterogeneity were again seen in all of these pooled analyses.

Table 4 lists the meta-regression results to find the factors associated with the means and LLNs of 2D-RVGLS and RVFWLS. Higher right ventricular FAC and GE Echopac vendor software (compared to other software) were associated with more negative means and LLNs of both RV strains. Right ventricular systolic pressure was also negatively associated with 2D-RVGLS mean and LLN but not 2D-RVFWLS mean or LLN. S3 Table in S1 Appendix showed the meta-regression results in GE EchoPAC studies only, which had similar findings for FAC and right ventricular systolic pressure, and also left ventricular global longitudinal strain to be positively correlated with both 2D-RVGLS mean and LLN but not 2D-RVFWLS.

**Table 4:** Meta-regression of two-dimensional right ventricular systolic strains mean and lower limit of normal.

	Mean			LLN		
Parameter	Beta	95% confidence interval	P-value	Beta	95% confidence interval	P-value
**Right ventricular global longitudinal strain**						
Year study published	0.05	-0.26, 0.35	0.768	0.08	-0.32, 0.49	0.685
Age (years)	0.06	-0.03, 0.14	0.196	**0.12**	**0.00, 0.23**	**0.046**
Male (%)	0.00	-0.11, 0.10	0.945	-0.02	-0.17, 0.13	0.830
Asian country (versus other)	0.42	-2.44, 3.27	0.776	1.03	-2.73,4.79	0.592
Body mass index (kg/m^2)	0.25	-0.25, 0.75	0.330	0.41	-0.23, 1.05	0.213
Body surface area (per 0.01 m^2)	-0.02	-0.23, 0.20	0.885	0.18	-0.04, 0.41	0.116
Heart rate (/minute)	0.14	-0.03, 0.32	0.111	0.13	-0.08, 0.34	0.217
Systolic blood pressure (mmHg)	0.06	-0.15, 0.26	0.577	0.26	0.00, 0.52	0.051
Left ventricular ejection fraction (%)	-0.13	-0.38, 0.13	0.335	-0.11	-0.42, 0.19	0.462
Left ventricular global longitudinal strain (%)	0.08	-1.00, 1.15	0.891	0.39	-0.88, 1.65	0.551
Right ventricular basal diameter (mm)	0.08	-0.07, 0.22	0.317	0.11	-0.11, 0.34	0.325
Right ventricular fractional area change	**-0.17**	**-0.30, -0.04**	**0.012**	**-0.31**	**-0.43, -0.18**	**<0.001**
Right ventricular S’ velocity (cm/s)	0.30	-0.61, 1.22	0.517	0.21	-1.10, 1.52	0.754
Tricuspid annular plane systolic excursion	-0.08	-0.59, 0.42	0.754	-0.39	-1.01, -0.24	0.227
Right ventricular systolic pressure	**0.76**	**0.21, 1.31**	**0.007**	**1.07**	**0.17, 1.97**	**0.020**
Echocardiography machine: Phillips versus GE	-0.50	-3.09, 2.08	0.703	1.08	-1.63, 3.79	0.434
Vendor: Not GE EchoPAC versus GE EchoPAC	**2.01**	**0.01, 4.02**	**0.048**	**3.90**	**1.73, 6.07**	**<0.001**
Frame rate (Hz)	-0.16	-0.42, 1.00	0.224	-0.17	-0.50, 0.17	0.335
**Right ventricular free wall longitudinal strain**						
Year study published	-0.09	-0.46, 0.28	0.632	-0.11	-0.56, 0.34	0.638
Age (years)	0.03	-0.08, 0.15	0.540	0.11	-0.02, 0.25	0.087
Male (%)	-0.01	-0.14, 0.11	0.830	-0.13	-0.28, 0.02	0.083
Asian country (versus other)	-1.20	-4.27, 1.86	0.441	-1.45	-5.17, 2.27	0.444
Body mass index (kg/m^2)	0.12	-0.57, 0.82	0729	0.56	-0.15, 1.26	0.121
Body surface area (per 0.01 m^2)	-0.08	-0.29, 0.13	0.439	0.22	-0.06, 0.57	0.120
Heart rate (/minute)	-0.09	-0.36, 0.15	0.49	-0.20	-0.45, 0.05	0.115
Systolic blood pressure (mmHg)	-0.15	-0.39, 0.10	0.240	0.01	-0.31, 0.33	0.962
Left ventricular ejection fraction (%)	0.00	-0.38, 0.56	0.955	0.30	-0.06, 0.67	0.100
Left ventricular global longitudinal strain (%)	0.81	-0.34, 1.95	0.166	**1.34**	**0.37, 2.31**	**0.007**
Right ventricular basal diameter (mm)	0.13	-0.27, 0.53	0.529	0.06	-0.29, 0.42	0.710
Right ventricular fractional area change	**-0.29**	**-0.43, -0.15**	**<0.001**	**-0.46**	**-0.63, -0.29**	**<0.001**
Right ventricular S’ velocity (cm/s)	0.70	-0.07, 1.54	0.088	0.80	-0.29, 1.89	0.151
Tricuspid annular plane systolic excursion	0.37	-0.27, 1.00	0.259	0.22	-0.39, 0.83	0.471
Right ventricular systolic pressure	0.35	-0.23, 0.93	0.239	0.74	-0.04, 1.53	0.061
Echocardiography machine: Phillips versus GE	-0.46	-3.19, 2.27	0.741	1.85	0.98 4.70	0.200
Vendor: Not GE EchoPAC versus GE EchoPAC	**2.29**	**0.27, 4.31**	**0.026**	**3.46**	**1.13, 5.78**	**0.004**
Frame rate (Hz)	0.00	-0.17, 0.16	0.969	0.00	-0.17, 0.17	0.975

## Discussion

This meta-analysis introduces novel definitions for abnormal, borderline and normal RV systolic systolic strain parameters, based on pooled LLNs and their 95%CI. For example, with RVGLS and its pooled LLN (95%CI) being -16.4% (-17.3%, -15.5%), a RVGLS measurement less negative than -15.5% would be considered abnormal, between -17.3% and -15.5% would be borderline, and more negative than -17.4% would be normal. We also provide for the first time pooled data for the LLNs of RVGLS, RVFWLS and IVSLS, pooled mean for RVGLS, separate data for 2D- and 3D- RV systolic strains and updated the pooled means for RVFWLS and IVSLS. These findings differed from the current echocardiography guidelines in terms on the value of the actual threshold (only defining that for RVFWLS at -20%), and adding the interval of borderline strain to the classification of abnormal and normal strains [1]. Furthermore, we identified important clinical and echocardiographic parameters that are associated with when measuring RV systolic strain that should be taken into account, such as RVFAC and vendor software.

Previous meta-analyses set out to report the normal values of strain in healthy subjects include for right ventricular strain by echocardiography [8], left ventricular strain by echocardiography [7, 55], and strain by magnetic resonance imaging [30]. These studies were each able to pool the means from studies of healthy subjects to provide the point estimates of mean strain parameters with very narrow 95%CIs that do not reflect the wider distribution of normal strains or provide estimates of LLN to distinguish normal and abnormal strains. In two recent meta-analysis for left ventricle strain by echocardiography and magnetic resonance imaging and this study, we overcome these limitations by estimating the LLNs of RV strains reported for eligible studies, i.e. 1.96 times the standard deviation plus the pooled mean, and calculating the SE of the LLN using Bland’s formula [9, 10, 12]. These estimates were then pooled using meta-analysis techniques to provide estimates of the LLN with 95%CI from multiple studies, with greater power and precision than which individual studies can provide.

The pooled LLNs and their respective 95%CIs reported in this study, rather than the pooled means, are necessary to defining reference ranges [9, 10]. The 95%CI of LLN reflects the range of values within which the true LLN is likely to lie. For example, the LLN of RVGLS and its corresponding 95% CI are -16.4% (-17.3%, -15.5%). This means that RVGLS values more negative than -17.3% is very likely normal, less negative than -15.5% is abnormal, while between -17.3% and -15.5% is indeterminate and therefore borderline. It should be noted that our findings reflect the reference range of RV systolic strain in healthy subjects, not thresholds of what is prognostically significant. In addition, the more narrow the 95%CI of the LLN the greater the reproducibility and robustness of the RV strain measurement. Therefore, pooled 3D-RV strain values we found with wider 95%CIs suggest that 3D techniques are currently less robust than 2D.

Our meta-regression analysis identified some important parameters associated with RV systolic strain. One important parameter is RVFAC, where a higher RVFAC was associated with more negative RVGLS and RVFWLS means and LLNs. This makes sense given that a higher RVFAC requires greater RV wall deformation and hence more negative strain, although perhaps a stronger association than expected given differences in RVFAC (angle-dependent) and RV strain measurement (angle-independent) techniques [1]. It is also notable that both tricuspid annular plane systolic excursion and right ventricular S’ tissue Doppler velocity which measures free wall function were not significant associated with especially RVFWLS. However, left ventricular ejection fraction was not associated with left ventricular strain in previous meta-analyses of healthy subjects [7, 55]. A future challenge would be to evaluate if RV systolic strain adds additional prognostic utility incrementally on other measures of RV systolic function. Of note, equations relating RVFAC or ejection fraction to RV systolic strains have not yet been developed, whereas one was recently published linking left ventricular ejection fraction with left ventricular strain using geometric modelling [56]. Another important parameter influencing RV systolic strain is right ventricular systolic pressure, analogous to the afterload of systolic blood pressure impacting on left ventricular systolic strain also previously reported [7].

RV systolic strains are also affected by the vendor software used to measure strain. GE Echopac continues to be the most widely used software in over 60% of the eligible studies, with other choices including TomTec, QLab, velocity vector imaging and Epislon and so they were combined for meta-regression analysis. Each software have their own unique method for strain analysis using speckle tracking outside the scope of this article, which may be vendor specific image acquisition such as EchoPAC for GE, or vendor independent such as velocity vector imaging. Our results showed that non-GE EchoPAC software had less negative means by 2–3% and LLNs by 4–5% for RVGLS and RVFWLS. Mixed results have been reported for the influence of vendor software on left ventricular strain using meta-regression, with borderline impact being reported with 2D- echocardiography (P = 0.08) [7], but significant impact when using 3D-echocardiography [55]. Individual studies with head-to-head strain measurements comparisons between vendor software are less commonly performed, have generally evaluated left ventricular longitudinal strain only, and often found small but statistically significance difference in strain values such as velocity vector imaging having less negative values than EchoPAC [10, 57]. Vendor software also needs to be taken into account when measuring and interpreting RV systolic strain according to this meta-analysis, and similar to left ventricular strain, if echocardiography is repeated, the same vendor software and version is preferred for serial comparisons of RV strain. These factors have important clinical implications to further “tailor” expected mean and LLNs to match individual patient strain measurements.

Even with identifying parameters that influence RV strain values in meta-regression, there remains considerable residual variability in normal RV strains reported in healthy subjects across studies. For example, the LLN for RVGLS in the study by Park et al was -15.2%, while it was -19.9% in that of Muraru et al, despite using the same software (GE Echopac) being used in both studies and similar sample sizes of >200 patients with narrow 95%CIs. The magnitude of these differences is difficult to explain. Potential causes are inter-institutional differences in data acquisition, software versions and analysis, as well as demographics. One way to circumvent this might be multicenter cooperation to retrospectively or quantify inter-laboratory variability, or prospective data collection to develop a definite reference sample for RV strain. What seems certain, however, is that it appears unlikely that any single institution would be able to provide a universally accepted reference range for RV strain parameters on its own.

Strain rates are the temporal derivative of strain, and are usually reported using their peak systolic or diastolic values [3].While systolic strain rate may offer additional information over strain measurement as it correlates with load independent measures of left [58] and right [59] ventricular contractility, it is seldom used in clinical practice. The main reason for this is that strain rate is more prone to signal noise than strain, and that it is influenced by temporal resolution of the ultrasound system. Nevertheless, we provide the pooled means and LLNs with 95%CIs for global, free wall and septal longitudinal strain rates for future research and clinical applications.

Like all meta-analyses, our study had some limitations. The accuracy of RV systolic strain measurement may differ by subject and study depending on operator experience and image quality. There was significant heterogeneity in the design and population (including patient characteristics and demographics, case-controlled studies), methodology of strain measurements (such as scanner and strain software vendor, technique at different laboratories), as well as the reported mean strains that were pooled as discussed earlier across studies, however this is similar to previous meta-analyses of strain [7, 8]. Further research is required to understand the heterogeneity of mean and LLN strain values across studies, especially as subgroup sensitivity analyses showed that these differences were not attributable to vendor software alone, to have a higher degree of confidence for applying pooled strain reference ranges into clinical practice and other studies. In the presence of duplication publication bias, we selected only the study with the largest number of healthy subjects, noting that there could still be residual bias by excluding these studies and patients. Not all clinical and echocardiographic characteristics were reported in all studies as seen in Tables 1 and 2, which can undermine univariable meta-regression findings and also meant we had insufficient power for multivariable meta-regression. We defined LLN arbitrarily as the boundary of the 95%CI consistent with other chamber quantification parameters [1], rather than based on prognostic significance in either healthy subjects or those with cardiovascular disease, so these findings should not be used for that purpose. The absence of patient-level data meant we had to use meta-analysis technique to calculate pooled means and reference ranges. Publication bias may be present although we did not find significant evidence for this. Nevertheless, most studies had high study quality according to the Newcastle-Ottawa quality assessment scale, and the criteria most commonly not fulfilled including source of control selection and comparability with cases were less relevant to the meta-analysis with our strict inclusion criteria for the definition of “healthy subjects”.

## Conclusion

In conclusion, this meta-analysis reports the reference ranges for RV systolic strains in healthy subjects by pooling means and more importantly LLNs with their 95%CIs. The latter allows the classification of RV systolic strains as normal, borderline and abnormal. For example, the pooled LLN for RVGLS was -16.4% (-17.3%, -15.5%), meaning that RVGLS less negative than -15.5% would be abnormal, between -17.3% and -15.5% borderline, and more negative than -17.3 normal. We obtained this reference range for RVGLS, RVFWLS and IVSLS measured by both 2D- and 3D speckle-tracking echocardiography. The most important factors associated with the means and LLNs of both RVGLS and RVFWLS measurements by meta-regression were RVFAC and vendor software which suggest a case could be made for separate reference ranges by vendor. Finally, caution must be applied when interpreting our findings given the significant heterogeneity of study populations in pooled analyses.

## Supporting information

S1 Checklist PRISMA checklist for this meta-analysis. (DOC)

S1 Appendix (PDF)
