# Supplementary material for: Defining the reference range for right ventricular systolic strain by echocardiography in healthy subjects: A meta-analysis
Source: PLoS One. 2021 Aug 20;16(8):e0256547. doi: 10.1371/journal.pone.0256547 (PMC8378693; doi:10.1371/journal.pone.0256547)
Supplement: S1 Appendix — (PDF) [file pone.0256547.s002.pdf]

S1 APPENDIX

Supplementary Appendix Table S1: Pooled means and lower limits of normal for right ventricular strain

Strain/vendor	Studies	N	Mean	95%CI (mean)	Heterogeneity testing	LLN	95%CI (LLN)	Heterogeneity testing
**EchoPAC (GE)**								
RVGLS (%)	19	2002	-23.9%	-24.8%, -22.9%	837 (<0.001), 97.9%	-17.5%	-18.3%, -16.6%	199 (<0.001), 90.9%
RVFWLS (%)	20	2050	-27.5%	-28.6%, -26.4%	652 (<0.001), 97.1%	-19.1%	-20.1%, -18.0%	192 (<0.001), 90.1%
**TomTec**								
RVGLS (%)	3	322	-23.6%	-25.2%, -22.0%	22 (<0.001), 90.8%	-15.2%	-17.0,% -13.4%	9.0 (0.011), 77.7%
RVFWLS (%)	3	618	-27.9%	-31.1%, -24.6%	78 (<0.001), 97.4%	-18.7%	-21.7%, -15.6%	51 (<0.001), 96.1%s
**Qlab**								
RVGLS (%)	0	N/A	N/A	N/A	N/A	N/A	N/A	N/A
RVFWLS (%)	3	408	-26.6%	-32.3%, -20.9%	288 (<0.001), 99.3%	-18.1%	-22.6%, -13.6%	71 (<0.001), 97.2%
**VVI**								
RVGLS (%)	2	236	-20.4%	-20.8%, -19.9%	0.21 (0.650), 0.0%	-11.3%	-17.1%, -5.6%	15 (<0.001), 93.2%
RVFWLS (%)	4	306	-24.2%	-26.6%, -21.8%	36 (<0.001), 91.6%	-14.2%	-17.8%, -10.6%	24 (<0.001), 87.6%

Abbreviations:

Supplementary Appendix Table S2: Pooled means and lower limits of normal for right ventricular strain in cohort studies with healthy subjects only (excludes case-controlled studies)

**Strain/vendor**	**Studies**	**N**	**Mean**	**95%CI (mean)**	**Heterogeneity testing**	**LLN**	**95%CI (LLN)**	**Heterogeneity testing**
RVGLS (%)	8	1408	-23.6%	-25.0%, -22.2%	474 (<0.001), 98.5%	-17.2%	-18.6,% -15.8%	159 (<0.001), 95.6%
RVFWLS (%)	9	2247	-26.6%	-28.6%, -24.7%	788 (<0.001), 99.0%	-17.0%	-19.6%, -14.4%	460 (<0.010), 98.2%

Supplementary Appendix Table S3: : Meta-regression of two-dimensional right ventricular systolic strains mean and lower limit of normal in studies utilizing GE-EchoPAC strain vendor software only

	Mean			LLN		
**Parameter**	**Beta**	**95% confidence interval**	**P-value**	**Beta**	**95% confidence interval**	**P-value**
**Right ventricular global longitudinal strain**						
Year study published	0.07	-0.23, 0.38	0.635	0.01	-0.24, 0.26	0.918
Age (years)	0.04	-0.07, 0.15	0.511	0.06	-0.05, 0.16	0.269
Male (%)	0.08	-0.04, 0.19	0.189	0.10	-0.01, 0.20	0.068
Asian country (versus other)	0.28	-0.283, 3.40	0.860	1.30	-1.07, 3.68	0.282
Body mass index (kg/m^2)	0.34	-0.41, 1.09	0.330	0.12	-0.60, 0.84	0.743
Body surface area (per 0.01 m^2)	-0.18	-0.40, 0.04	0.117	-0.06	-0.26, 0.14	0.551
Heart rate (/minute)	0.15	-0.02, 0.32	0.082	0.12	-0.02, 0.27	0.096
Systolic blood pressure (mmHg)	-0.15	-0.44, 0.14	0.311	-0.09	-0.34, 0.16	0.482
Left ventricular ejection fraction (%)	-0.07	-0.32, 0.182	0.590	0.07	-0.07, 0.22	0.321
Left ventricular global longitudinal strain (%)	**1.15**	**0.16, 2.13**	**0.023**	**1.10**	**0.62, 1.58**	**<0.001**

Right ventricular basal diameter (mm)	0.08	-0.04, 0.19	0.197	0.04	-0.08, 0.17	0.509
Right ventricular fractional area change	**-0.49**	**-0.87, -0.12**	**0.010**	**-0.45**	**-0.75, -0.15**	**0.004**
Right ventricular S' velocity (cm/s)	0.43	-0.25, 1.11	0.211	0.10	-0.23, 0.43	0.561
Tricuspid annular plane systolic excursion	-0.11	-0.61, 0.41	0.651	**-0.47**	**-0.84, -0.10**	**0.013**
Right ventricular systolic pressure	**0.62**	**0.22, 1.10**	**0.002**	**0.45**	**0.05, 0.84**	**0.026**
Frame rate (Hz)	-0.21	-0.46, 0.04	0.095	**-0.24**	**-0.36, -0.12**	**<0.001**
**Right ventricular free wall longitudinal strain**						
Year study published	-0.01	-0.45, 0.43	0.959	-0.15	-0.57, 0.27	0.483
Age (years)	-0.02	-0.18, 0.14	0.817	0.05	-0.11, 0.21	0.514
Male (%)	-0.08	-0.23, 0.07	0.275	-0.05	-0.19, 0.09	0.458
Asian country (versus other)	-1.43	-4.64, 1.78	0.382	-1.74	-4.81, 1.34	0.269
Body mass index (kg/m^2)	0.74	-0.29, 1.76	0.154	**1.14**	**0.10, 2.81**	**0.032**
Body surface area (per 0.01 m^2)	0.00	-0.22, 0.23	0.988	0.09	-0.12, 0.32	0.383
Heart rate (/minute)	-0.02	-0.27, 0.24	0.888	-0.05	-0.30, 0.20	0.677
Systolic blood pressure (mmHg)	-0.16	-0.41, 0.10	0.230	0.02	-0.27, 0.32	0.882
Left ventricular ejection fraction (%)	0.18	-0.22, 0.57	0.387	0.26	-0.07, 0.60	0.123

Left ventricular global longitudinal strain (%)	0.40	-1.65, 2.44	0.702	0.61	-0.43, 1.65	0.247
Right ventricular basal diameter (mm)	0.07	-0.49, 0.62	0.819	-0.19	-0.63, 0.25	0.399
Right ventricular fractional area change	**-0.41**	**-0.56, -0.27**	**<0.001**	**-0.24**	**-0.42, -0.07**	**0.007**
Right ventricular S’ velocity (cm/s)	0.83	-0.42, 2.07	0.192	**1.23**	**0.22, 2.24**	**0.017**
Tricuspid annular plane systolic excursion	0.27	-0.53, 1.06	0.514	0.26	-0.50, 1.02	0.502
Right ventricular systolic pressure	0.32	-0.30, 0.93	0.315	0.44	-0.15, 1.04	0.146
Frame rate (Hz)	0.150	-0.04, 0.34	0.128	**0.22**	**0.01, 0.44**	**0.045**
